# The hardships of the poorest during the COVID-19 pandemic: Data about the socioeconomic conditions and governance of informal workers

**DOI:** 10.1016/j.dib.2021.107728

**Published:** 2021-12-16

**Authors:** Lina Martínez, Grame Young, Valeria Trofimoff, Isabella Valencia, Nicolás Vidal, Andrés David Espada, Esteban Robles

**Affiliations:** a Universidad Icesi – POLIS^1^, Cali, Colombia; b Center for Sustainable, Healthy and Learning Cities and Neighbourhoods, University of Glasgow, UK

**Keywords:** COVID-19, Informal workers, Socioeconomic conditions, Governance, Social policy, Colombia

## Abstract

This report presents survey data about the socioeconomic conditions and governance of informal workers in Cali, Colombia during the COVID-19 pandemic. Conducted with 750 street vendors via telephone, the survey explores eight interrelated topics: demographics, households and children, economic activities, income and expenses, access to financial services and debt, institutional trust, health, and subjective wellbeing. These data are valuable for two reasons. First, they allow for an analysis of the social and economic consequences of the pandemic for a population group that remains understudied and neglected by social policy. Second, they allow for an understanding of the governance of informal work during crises and possible paths to promote greater inclusion. Taken together, the data presented here provide tools for conducting academic and policy-relevant analysis about informal workers, the long-term consequences of COVID-19 in the global South, and how recovery from the pandemic can be made more inclusive and sustainable.

**Specifications Table**

**Table:** 

Subject area	Social Science
More specific subject area	*Social Policy – Poverty reduction*
Type of data	Text, dummy, and metric variables
How data were acquired	Phone surveys
Data format	Raw
Parameters for data collection	Adult informal workers from Cali Colombia contacted by telephone. Cellphone number was provided to researchers by association leaders.
Description of data collection	Phone survey of adult informal workers in Cali, Colombia. For data collection, researchers approached leaders of workers’ associations and asked for voluntary participation in the survey. Leaders of workers’ associations collected cellphone numbers of street vendors willing to participate in the study. Trained pollsters contacted informal workers by phone, and respondents referred pollsters to other potential respondents. A web platform was created for the study: https://www.icesi.edu.co/polis/sppagebuilder.php?id=131&view=page
Data source location	Institution: POLIS – Observatorio de políticas públicas – Universidad Icesi City/Town/Region: Cali - Valle del Cauca Country: Colombia Contact email: polisicesi@icesi.edu.co
Data accessibility	Available at Mendeley Data [1]. https://data.mendeley.com/datasets/w5×3dp8t4z/1

## Value of the Data

• The data presented here provide important insights into the experiences and perspectives of street vendors in a mid-sized city in the global South during the COVID-19 pandemic. As informal workers, street vendors face significant livelihood challenges and forms of insecurity. This unique dataset elucidates these challenges and insecurities in the context of a major public health crisis with significant socioeconomic consequences and highlights potential ways in which they can be addressed.
• The dataset described in this article has considerable valuable for academics studying street vending, informal work, urban development, and the experiences and perspectives of the urban poor during the COVID-10 pandemic; policymakers who aim to address the challenges that street vendors face in relation to a broad range of socioeconomic issues; and civil society groups engaged in research and advocacy activities aimed at improving the quality of life of street vendors and informal workers more generally.
• The data can be used/reused to develop comparative insights into the experiences and perspectives of street vendors, and those of other informal workers, across the global South during the COVID-19 pandemic. They can also be used in future assessments of the extent to which post-pandemic recovery efforts are meeting the needs and serving the interests of street vendors and of informal workers and other marginalized groups.

## 1 Data Description

### 1.1 Questionnaire and variables

This aims to measure the social, economic, and political implications of the COVID-19 pandemic for street vendors in Cali, the third-largest city in Colombia. The questions were primarily drawn from national household surveys [2] and previous studies conducted in the city about informality and street sales [3,4], with additional questions designed by the lead researchers on the project.

The questionnaire has eight sections: (i) demographic data; (ii) household and children; (iii) economic activity; (iv) income and expenses; (v) access to financial services and indebtedness; (vi) institutional trust; (vii) health; and (viii) subjective wellbeing.

***Demographic data***. The information in this section describes the participants and allows for focused analysis based on sociodemographic characteristics. Participants were asked about age, gender, neighborhood of residency, household strata (according to the national socioeconomic status (SES) scale to classify households, in which households in category SES 1 are the poorest and SES 6 are the most affluent (according to census categories)), race/ethnicity, household ownership, type of health insurance, and contribution to health and retirement plans. Table 1 presents descriptive statistics from this section.

**Table 1:** Socioeconomic variables.

Demographic data	
Average age (years)	51
Female (%)	49
**SES (%)**	
SES 1	43
SES 2	37
SES 3	19
SES 4	1
SES 5	0
SES 6	0
**Ethnicity (%)**	
White	22
Multi-racial	41
Native	6
Black/Afro	25
Other	5
None	2
**Type of household (%)**	
Own paying (mortgage)	5
Own paid	21
Rented	46
Family	19
Rented room	7
Other	1
**Health insurance scheme (%)**	
Contribute	11
Subsidized	63
Beneficiary	14
Special	1
None	9
DK	2
**Contribute to health and pension (%)**	
Only to health	10
Only pension	1
Both	3
None	85
Pensioner	1

***Household and children*.** This section inquires about household composition, measured by the number of members in the household, the number of children, and whether the respondent was the household head (primary income earner). In this section, the survey also includes variables for children attending school and dropping out during the pandemic. Likewise, a question about children having access and the access frequency to a computer or electronic device, the internet, and food is included to analyze the conditions faced by street vendors’ children during the pandemic. Table 2 presents general information about this set of variables.

**Table 2:** Household and children.

**Household and children**	
Head of household (%)	73
Average people living in the household	4
Average number of children	2
**Children dropped out of school during pandemic (%)**	
Yes	9
No	31
No school-age children	60
**Children always have access to (%)**	
Computer or electronic device	66
Internet	63
Food	86

**Table 3:** Economic activity.

**Economic activity**	
Average years working as street vendor	19
Satisfied with your current occupation (%)	58
**Before the pandemic, how did you consider your income? (%)**	
Good (enough to cover basic needs and save)	56
Regular (enough to just cover basic needs)	42
Bad (could not cover basic needs)	2
**Days without work during the pandemic (%)**	
Less than a month (30 days)	4
Between one and two months (30–60 days)	22
More than 3 months (90+ days)	74
I did not lose any working days (0 days)	0
**Average work schedule**	
Average days worked per week	6
Average hours worked per day	9

***Economic activity****.* This section explores the amount of time in years respondents have worked as a street vendor, their general satisfaction with their occupation, income reduction during the pandemic, their income prior to the pandemic, the average time street vendors were out of work during the lockdown imposed in the city, and the number of days each week and hours each day there were able to work. This set of questions, combined with those included in the next section, provides important insights into the economic effects of the pandemic for street vendors (Table 3).

***Income and expenses****.* Questions in this section collect information about average monthly sales, average monthly profit, perception of sales providing enough resources to cover livelihood expenses, income reduction during the pandemic, compensating for income reduction, and income perception (good, regular, bad). Table 4 presents this set of variables.

**Table 4:** Income and expenses.

**Income and expenses**	
Average monthly sales (USD)*	369
Average monthly profit (USD)*	145
**Does your business provide you with sufficient resources for your livelihood? (%)**	
Yes	18
No	36
Sometimes	45
DK	1
**Has your income been reduced due to the pandemic?**	
Your income has reduced (%)	97
**How did you compensate for the reduction in your income? (%)**	
Developing another economic activity	28
Drawing on your savings	28
Asking for help from family or friends	50
Getting into debt	20
Receiving financial support from the state (subsidies)	12
Reducing expenditures	29
Other	2
**Currently, how do you consider your income? (%)**	
Good (enough to cover basic needs and save)	4
Regular (enough to just cover basic needs)	49
Bad (could not cover basic needs)	47

⁎ 1 US dollar = 3400 Colombian peso (average currency - 2021).

***Access to financial services and debt****.* This set of questions aims to offer insights into the financial hardships of street vendors during the pandemic. Respondents were asked about debts/loans before the pandemic and whether they had to apply for a loan/credit during the pandemic. In cases where respondents indicated accessing a loan in the last year, the questionnaire includes a set of questions to analyze the loan characteristics, focusing on lender type, average interest rate, loan amount, monthly installment, and loan duration. The survey also asks about the purpose of the loan, whether street vendors repaid past loans, and difficulties paying debts.

These questions allow for an analysis of the financial exclusion of informal workers and the mechanisms to access credit during the pandemic. Likewise, the questions serve as a proxy to study the penetration of informal and unregulated types of credit like payday loans in the informal sector. Results of this set of questions are reported in Table 5.

**Table 5:** Access to financial services and debt.

**Access to financial services and debt**	
Before the pandemic you have debts or loans (%)	33
During the pandemic you applied for a loan (%)	21
Of respondents who applied for a loan during the pandemic*	
Loan with family (%)	19
Loan with friends (%)	19
Loan with a bank (%)	24
Loan with pay-day lender (%)	29
Loan with microfinance institution (%)	5
Average monthly interest rate (%)	8
Amount of last loan (USD)⁎⁎	290
Amount of last installment (USD)⁎⁎	30
Debt term (months)	8
**Objectives of loans (%)**	
Business	55
Debts	31
Recreation	0
Health	2
Housing	38
Payment of public services	34
Education	2
Other	17
**Did you pay the last loan you made? (%)**	
Yes	37
No	3
In payment process	60
**Trouble paying debts (%)**	
Have difficulties paying debts (%)	77

⁎ All subsequent figures in this table refer to respondents who applied for a loan during the pandemic.

⁎⁎ 1 US dollar = 3400 Colombian peso (average currency - 2021).

***Institutional trust, welfare programs, and political participation****.* This section has 13 questions to proxy for different measures of street vendors’ perceptions of and relationship with the local government. Three questions are included to measure institutional trust (trust on the city council, national police, and public officials), measured on a scale from 0 to 10. These questions were designed by the Organization for Economic Co-operation and Development (OECD) to be included in population surveys [5]. Another set of questions refers to access to welfare programs like subsidies, street vendors’ views on government support, and other key issues surrounding informal work. The questionnaire also includes questions about the perception of government performance and communication with street vendors in terms of restrictions during the pandemic. The survey includes questions about participation in associations and respondents’ perception of whether belonging to an association increase political participation to serve as a proxy for political participation. Table 6 presents the results of the questions included in this section.

**Table 6:** Institutional trust, welfare programs, and political participation.

Institutional trust	
**Institutional trust (average scale 0–10)**	
Municipal Council	3.9
Police	4.1
Civil service	4.1
**Support from government (%)**	
Feel support from the government	21
Beneficiary of any subsidy and/or benefit promoted by the State	23
The government has been clear with the opening of the places and the dates in which you can carry out your work	47
**What programs should the government focus on? (%)**	
Finance inclusion programs	26
Job-training programs	30
Education programs	24
Relocation	14
Increasing formal employment	37
Subsidies for housing	46
Subsidies - compensatory income	64
Food	61
Regulations to allow informal workers to continue working in their current occupations	48
Other	3
**Do you agree with the following statements? (average scale 1–5)**	
I am able to find formal employment	1.7
I would prefer formal employment	3.4
I would prefer running a formal business	3.9
The pandemic has had a negative impact on the job opportunities that are available to me	3.9
I can easily access the spaces I need to conduct my business	3.3
Government regulations make it more difficult to conduct my business	3.8
**Access to public programs (%)**	
Job placement	0
Education and new skills	1
Unemployment insurance	1
Public housing	3
Cash transfers	18
School access for children*	39
**Harassment by the police (%)**	
Increased	22
Decreased	18
No victim	60
**Government satisfaction**	
Satisfaction with the municipal government's management of pandemic (average scale 0–10)	4.6
**Informal workers’ associations (%)**	
Belong to an informal workers’ association	70
Believe that belonging to an informal workers’ association increases political participation	39

⁎ Calculated for street vendors with school-age children.

***Health****.* The survey includes five questions that are designed to explore the general health status of street vendors, their households, and others close to them. Questions examine whether the respondent or someone in their household got sick from COVID-19 or another illness; whether the sick person in the household was able to access medical care; if the respondent lost someone as a consequence of COVID-19; and lastly, if the respondent or someone in their household went to bed hungry during the pandemic. Table 7 presents the general results.

**Table 7:** Health.

**Health**	
You or someone in the household with COVID-19 or other illness (%)	26
Ill by COVID-19 (%)*	58
Able to access health services (%)*	
Yes	53
No	12
No need of medical attention (%)	35
**Lost family member or anyone close to you (%)**	
Lost someone as consequence of the pandemic (%)	34
**Hunger (%)**	
You or someone in the household have gone to bed hungry during the pandemic	21

⁎ All questions with an asterisk refer to respondents who reported they or someone in their household was ill with COVID-19 or another illness.

***Subjective wellbeing****.* The survey includes five questions to measure subjective wellbeing, measured on a scale from 0 to 10. Four questions—life satisfaction and how happy, worried, and depressed the respondent felt in the previous day—come from the core measure of subjective wellbeing suggested by the OECD to be included in household surveys [6]. One question about anxiety is included in the survey using the same scale. Table 8 presents the general results of this set of questions.

**Table 8:** Subjective wellbeing.

**Subjective wellbeing**	
Life satisfaction (average scale 0–10)	6.9
Increase on anxiety and stress levels in last few days (average scale 0–10)	6.8
How happy you felt yesterday (average scale 0–10)	6.2
How worried you felt yesterday (average scale 0–10)	6.3
How depressed you felt yesterday (average scale 0–10)	3.4

## 2 Experimental Design, Materials and Methods

The survey was administered between March and May 2021 as part of a research project, financed by the Center for Sustainable, Healthy, and Learning Cities and Neighborhoods, entitled “Promoting Inclusive Governance for Informal Workers in Cali, Colombia”. Given the restrictions imposed by COVID-19, it was not possible to conduct the survey through face-to-face interviews, so it was instead conducted via telephone. To do this, researchers in Cali reached out to the leaders of street vendors’ associations (15 in total), explained the purpose of the study, and requested the participation of association members. Leaders communicated the purpose of the study to their association members and began collecting phone numbers of individuals who were willing to participate, which were given to researchers to contact respondents, who then referred pollsters to additional potential respondents. Participants gave their consent to use the information collected in the study for academic purposes. No personal information (name, ID number, address, or working location) was asked to assure confidentiality. The phone survey typically lasted about 20 min, although in some instances this was considerably longer given business interruptions. Researchers had a 50% response rate.

## 3 Piloting and Data Access

The questionnaire was piloted before implementation with four street vendors. Minor adjustments were made to the survey after the pilot. Annex A presents the complete questionnaire used in this study. The raw data and the survey can be accessed through the MendeleyData [1] repository.

## Ethics Statement

The ethics committee of Universidad Icesi approved the surveys before implementation (code # 348), respondents provided consent for the information to be used for academic purposes, and participation was voluntary. The survey did not include any experimentation with human subjects.

## CRediT authorship contribution statement

**Lina Martínez:** Conceptualization, Funding acquisition, Data curation, Formal analysis, Methodology, Writing – review & editing, Supervision. **Grame Young:** Methodology, Funding acquisition, Writing – review & editing. **Valeria Trofimoff:** Data curation, Formal analysis. **Isabella Valencia:** Data curation, Formal analysis. **Nicolás Vidal:** Data curation, Formal analysis. **Andrés David Espada:** Data curation, Formal analysis. **Esteban Robles:** Data curation, Formal analysis.

## Declaration of Competing Interest

The authors declare that they have no known competing financial interests or personal relationships that could have influenced the work reported in this paper.

## Appendix Supplementary materials

Image, application 1
