# Corrigendum to ‘The hardships of the poorest during the COVID-19 pandemic: Data about the socioeconomic conditions and governance of informal workers’ *Data in Brief* [Volume 40, February 2022, 107,728]

**DOI:** 10.1016/j.dib.2022.108184

**Published:** 2022-04-12

**Authors:** Lina Martínez, Graeme Young, Valeria Trofimoff, Isabella Valencia, Nicolás Vidal, Andrés David Espada, Esteban Robles

**Affiliations:** a Universidad Icesi – POLIS, Cali, Colombia; b Centre for Sustainable, Healthy and Learning Cities and Neighbourhoods, University of Glasgow, United Kingdom

The authors regret that the previous version of this article misspelled Graeme Young's name.

The authors would like to apologize for any inconvenience caused.

