# Supplementary material for: The hardships of the poorest during the COVID-19 pandemic: Data about the socioeconomic conditions and governance of informal workers
Source: Data Brief. 2021 Dec 16;40:107728. doi: 10.1016/j.dib.2021.107728 (PMC8699104; doi:10.1016/j.dib.2021.107728)
Supplement: Supplementary file 1 [file mmc1.pdf]

ICESI / POLIS	STREET VENDORS DURING THE PANDEMIC	2021

This survey is anonymous and does not collect personal data. Consequently, it is not possible to identify you. The information is collected for strictly academic purposes. Thank you for your participation.

DEMOGRAPHIC DATA

1. Birth year

____

2. Gender

1 ☐ Male
2 ☐ Female

3. Which neighborhood do you live in?

____

4. Which is the socio-economic strata of your household?

1 ☐
2 ☐
3 ☐
4 ☐
5 ☐
6 ☐

5. According to your cultural, ethnic group or physical characteristics, how do you recognize yourself?

1 ☐ White
2 ☐ Multi-racial
3 ☐ Native
4 ☐ Black/Afro
5 ☐ Other
6 ☐ None

6. Your household is:

1 ☐ Own paying (mortgage)
2 ☐ Own paid
3 ☐ Rented
4 ☐ Family
5 ☐ Inquilinato
6 ☐ Another, which one? ________________

7. Which health insurance scheme are you affiliated?

1 ☐ Contribute
2 ☐ Subsidized
3 ☐ Beneficiary
4 ☐ Special
5 ☐ None
6 ☐ DK

8. Do you contribute to health and pension?

1 ☐ Only to health
2 ☐ Only pension
3 ☐ Both
4 ☐ None
5 ☐ You are a pensioner
6 ☐ DK

HOME AND CHILDREN

9. Are you head of household?

1 ☐ Yes
2 ☐ No

10. Including yourself, how many people live in your household?

____

11. How many children do you have? ***If you do not have children, go to the next section***

0	1	2	3	4	5	More than 5

12. Any of your school age children dropped from school during the past year ?

1 ☐ Yes
2 ☐ No
3 ☐ Does not apply, if you do not have school-age children

13. Your children have had access to the following tools studying at home:

		Always	Sometimes	Never
1	Computer or electronic device			
2	Internet			
3	Food			

ECONOMIC ACTIVITY

14. How long have you been working as a street vendor?

____

1 ☐ Months
2 ☐ Years

15. Are you satisfied with your current occupation?

1 ☐ Yes
2 ☐ No
3 ☐ DK

16. Before the pandemic, how did you consider your income?

1 ☐ Good (enough to cover basic needs and save).
2 ☐ Regular (they were enough to just cover the basic needs).
3 ☐ Bad (they could not cover basic needs).

17. How many days have you been unemployed during the quarantine?

1 ☐ Less than a month (30 days)
2 ☐ Between one and two months (30-60 days)
3 ☐ More than 3 months (+90 days)
4 ☐ I did not lose any working days (0 days)

CONTINUE

18. On average, how many days per week can you work during the current crisis?

Days ___________

19. On average, how many hours do you work per day?

Daily hours ___________

INCOME AND EXPENSES

20. Currently, how much are your daily sales on average?

Daily ______________________

21. Currently, how much are your daily earnings on average?

Daily ______________________

22. Does your business provide you with sufficient resources for your livelihood?

1 [ ] Yes
3 [ ] Sometimes
2 [ ] No
4 [ ] DK

23. Has your income been reduced due to the pandemic?

1 [ ] Yes
2 [ ] No (Go to q25)

24. How did you compensate for the reduction in your income? (multiple choice)

1 [ ] Developing another economic activity
2 [ ] Drawing on your savings
3 [ ] Asking for help from family or friends
4 [ ] Getting into debt
5 [ ] Receiving financial support from the state (subsidies)
6 [ ] Reducing expenditures
7 [ ] Other, which one? ________________

25. Currently, how do you consider your income?

1 [ ] Good (enough to cover basic needs and save).
2 [ ] Regular (they were enough to just cover the basic needs).
3 [ ] Bad (they could not cover basic needs).

ACCESS TO FINANCIAL SERVICES AND DEBT

26. Before de pandemic, did you have any debts or loans?

1 [ ] Yes
2 [ ] No

27. During the pandemic, have you applied for a loan?

1 [ ] Yes
2 [ ] No (Go to q36)

28. With whom did you make the loan?

1 [ ] Family
2 [ ] Friend
3 [ ] Bank
4 [ ] Day per day loan
5 [ ] Microfinance institution
6 [ ] Other

29. What was the monthly interest rate on the credit you purchased?

____

30. What is the amount of the last loan you applied for and / or requested?

$ ________________________________

99 [ ] Do not know not answer

31. How much is the last installment you paid?

$ ________________________________

99 [ ] Do not know not answer

32. What was the length of time that you got into debt during the last loan?

____

1 [ ] Days
2 [ ] Months
3 [ ] Years

33. What were the objectives of the loans you made? (MULTIPLE ANSWER)

1 [ ] Business
2 [ ] Debts
3 [ ] Recreation
4 [ ] Health
5 [ ] Housing
6 [ ] Payment of public services
7 [ ] Education
8 [ ] Other, which one?

____

34. Did you pay the last loan you made?

1 [ ] Yes
2 [ ] No
3 [ ] In payment process

35. Are you having trouble paying your debts?

1 [ ] Yes
2 [ ] No
3 [ ] You have no debts

INSTITUCIONAL TRUST

The next questions are about your level of trust in various institutions in the city. Even if you have had little or no contact with these institutions. Please base your answers on your general impression of these institutions. Please answer on a scale of zero to ten, where zero means not at all and 10 means always

36. The Municipal Council?

Not trust at all | 0 | 1 | 2 | 3 | 4 | 5 | 6 | 7 | 8 | 9 | 10 | Trust at all

37. The National Police?

Not trust at all | 0 | 1 | 2 | 3 | 4 | 5 | 6 | 7 | 8 | 9 | 10 | Trust at all

38. Civil service?

Not trust at all | 0 | 1 | 2 | 3 | 4 | 5 | 6 | 7 | 8 | 9 | 10 | Trust at all

39. Do you feel support from the government regarding the economic situation of your home?

1 ☐ Yes   2 ☐ No

40. Are you a beneficiary of any subsidy and / or benefit promoted by the State? (Families in Action, Colombia Mayor, Solidarity Income or other.)

1 ☐ Yes   2 ☐ No

41. Has the government been clear with the opening of the places and the dates in which you can carry out your work?

1 ☐ Yes   2 ☐ No

42. In your opinion, what kind of programs should be the goverment priority to aliviate the current conditions of informal workers?

1 ☐ Finance inclusion programs
2 ☐ Work- training programs
3 ☐ Education programs
4 ☐ Relocation
5 ☐ Increasing formal employment
6 ☐ Subsidies for housing
7 ☐ Subsidies - compensatory income
8 ☐ Food
9 ☐ Regulations to allow informal workers continue working on their current occupation
10 ☐ Other, which one? ________________

43. Do you agree with the following statements?

1. I am able to find formal employment	Disagree	1	2	3	4	5	Agree
2. I would prefer formal employment	Disagree	1	2	3	4	5	Agree
3. I would prefer running a formal business	Disagree	1	2	3	4	5	Agree
4. The pandemic has had a negative impact on the job opportunities that are available to me	Disagree	1	2	3	4	5	Agree
5. I can easily access the spaces I need to conduct my business	Disagree	1	2	3	4	5	Agree
6. Government regulations make it more difficult to conduct my business	Disagree	1	2	3	4	5	Agree

44. Do you have access to any of the following programs?

	Yes (1)	No (2)
1. Job placement programs		
2. Education develop skills for a new job		
3. Employment insurance		
4. Government provided social housing		
5. Monetary subsidies		
6. Affordable, good quality public schools for children		

45. You consider that during the pandemic the police persecution towards your business has:

1 ☐ Increased   2 ☐ Decreased   3 ☐ You have not been a victim of police persecution

46. Overall, how satisfied are you with the government's management during the pandemic?

Not satisfied | 0 | 1 | 2 | 3 | 4 | 5 | 6 | 7 | 8 | 9 | 10 | Very satisfied

47. Do you belong to an informal workers association?

1 ☐ Yes   2 ☐ No (Go to q49)

48. Do you consider that belonging to a workers association increase the political participation?

1 ☐ Yes   2 ☐ No

UNIVERSIDAD ICESI POLIS	STREET VENDORS DURING THE PANDEMIC	2021

HEALTH

49. Since the pandemic started, have you or someone in your household gotten sick from COVID-19 or some other disease?

1 ☐ Yes   2 ☐ No (Go to q52)

51. If you or a family member have gotten sick, have you been able to go to a medical center?

1 ☐ Yes   2 ☐ No   3 ☐ No need medical attention

50. Did you get sick with Covid-19 or another disease?

1 ☐ Covid-19
2 ☐ Another disease

52. Have you lost a family member or close person as a result of the pandemic?

1 ☐ Yes   2 ☐ No

53. Have you or someone in your household gone to bed hungry during the pandemic?

1 ☐ Yes   2 ☐ No

SUBJECTIVE WELL-BEING

54. In general, how satisfied are you with all aspects of your life?

Not satisfied	0	1	2	3	4	5	6	7	8	9	10	Very satisfied

55. Do you feel that in the last few days your anxiety and stress levels have increased?

Completely desagree	0	1	2	3	4	5	6	7	8	9	10	Completely agree

The next questions are about how you felt yesterday on a scale of 0 to 10. Zero means that you did not experience these feelings "at any time" while 10 means that you experienced these feelings "all the time." Now I am going to read you a list of scenarios that you could experience yesterday.

In any moment	0	1	2	3	4	5	6	7	8	9	10	All the time

**56.1 How happy?**	0	1	2	3	4	5	6	7	8	9	10
**56.2 How worried?**	0	1	2	3	4	5	6	7	8	9	10
**56.3 How depressed?**	0	1	2	3	4	5	6	7	8	9	10

END OF SURVEY
